# Supplementary material for: SNPtotree—Resolving the Phylogeny of SNPs on Non-Recombining DNA
Source: Genes (Basel). 2023 Sep 22;14(10):1837. doi: 10.3390/genes14101837 (PMC10606150; doi:10.3390/genes14101837)
Supplement: Supplementary file 1 [file genes-14-01837-s001.zip › Supplementary_Files/SNPtotree_SupplementaryMaterial1.pdf]

SNPtotree – Supplementary Material 1

Supplementary Material 1.1: Filtered recurrent and backmutated variants

SNPtotree strictly filters variants due to contradictory relationships or ambiguous positions of the variants in the tree. The variants filtered out in any of the two filtering steps could be the result of sequencing errors, but possibly also recurrent mutations and backmutations.

Recurrent mutations describe the same mutation occurring twice during evolution, originating two alleles that are identical by state, rather than by descent, like in convergent evolutionary traits. In a phylogenetic tree, recurrent mutations can be observed as the same variant allele on different branches. The presence of recurrent mutations may lead to contradictory relationships between two non-allelic variants and/or an ambiguous position in a phylogenetic tree. However, if the derived allele is present in all informative sequences, it may not be possible to ascertain the recurrency of the mutation and the variant may not be filtered out.

Backmutations describe a second mutational event at the same base position that restores the ancestral allele. Palindromic regions, commonly observed in the human Y chromosome, enable gene conversions and thus backmutations. Backmutations will most often result in contradictory relationships.

The recurrent and backmutated variants removed in the software can be saved in an additional output file, and manually analyzed, as by constructing maximum parsimony trees.

Supplementary Material 1.2: RAxML settings

With default settings, 20 maximum likelihood trees were generated (1). Next, support values were calculated for 100 trees by conducting bootstrap analysis (2), and finally the bootstrap values were applied on the best maximum likelihood tree (3).

(1) *./raxmlHPC-PTHREADS-AVX2 -m ASC_GTRGAMMA --asc-corr lewis -s /path_to_fasta_file.fasta -T 60 -n output_name_T1 -# 20 -p 54023*

(2) *./raxmlHPC-PTHREADS-AVX2 -m ASC_GTRGAMMA --asc-corr lewis -s /path_to_fasta_file.fasta -T 60 -n output_name_T1_BS -# 100 -p 54023 -b 54023*

(3) ./raxmlHPC-PTHREADS-AVX2 -m ASC_GTRGAMMA --asc-corr lewis -T 60 -n output_name_Best+BS -p 54023 -f b -t RAxML_bestTree.output_name_T1 -z RAxML_bootstrap. output_name_T1_BS
