# Supplementary material for: SNPtotree—Resolving the Phylogeny of SNPs on Non-Recombining DNA
Source: Genes (Basel). 2023 Sep 22;14(10):1837. doi: 10.3390/genes14101837 (PMC10606150; doi:10.3390/genes14101837)
Supplement: Supplementary file 1 [file genes-14-01837-s001.zip › Supplementary_Files/Supplementary_Figures1-4.pdf]

Supplementary Figures 1 - 6

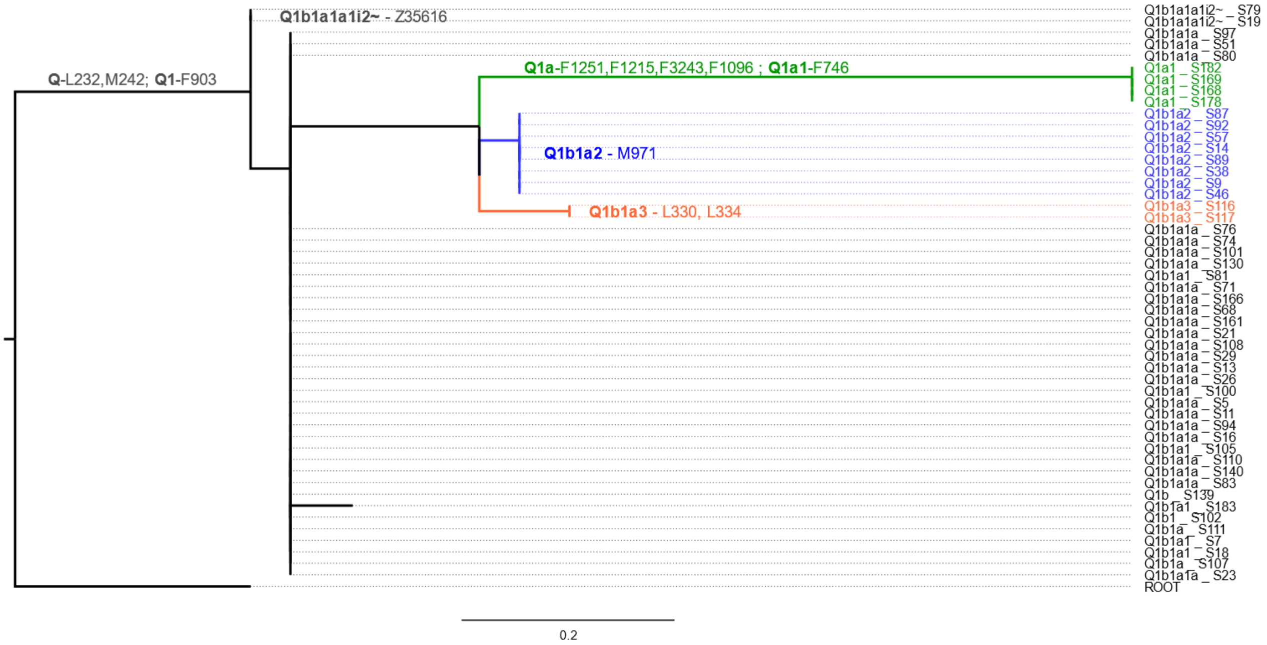

Supplementary Figure 1: Maximum likelihood phylogeny of the Y-chromosomal clade Q presented in testdata 1. The tip labels present one individual each and are composed of the highest resolution (or most downstream) clade and the sequence name. Clades and tip labels highlighted in colors present clearly separated sub-lineages. SNPs shared only by sequences of certain clades are labelled at the respective clade branch or node. The tree was manually rooted with an ancestral outgroup (sample HGDP00449).

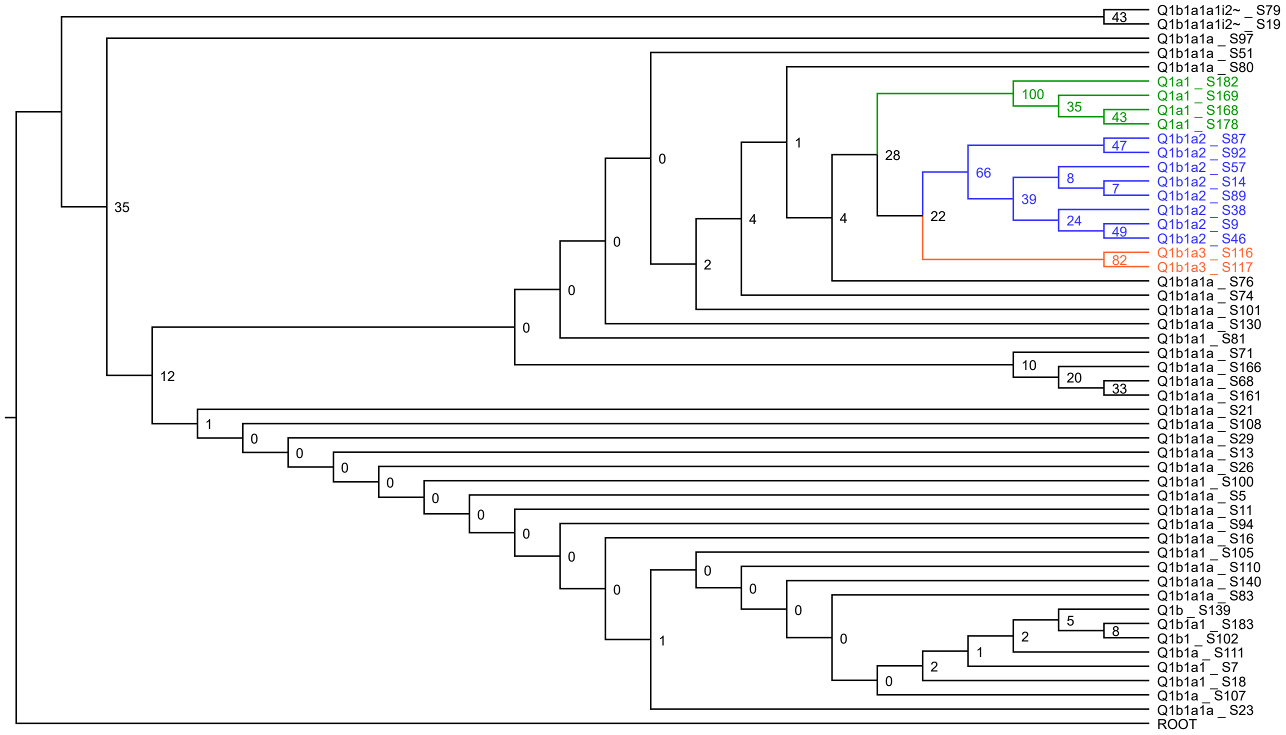

Supplementary Figure 2: Maximum likelihood phylogeny of the Y-chromosomal clade Q from testdata 1 shown as a cladogram to present the bootstrap values.

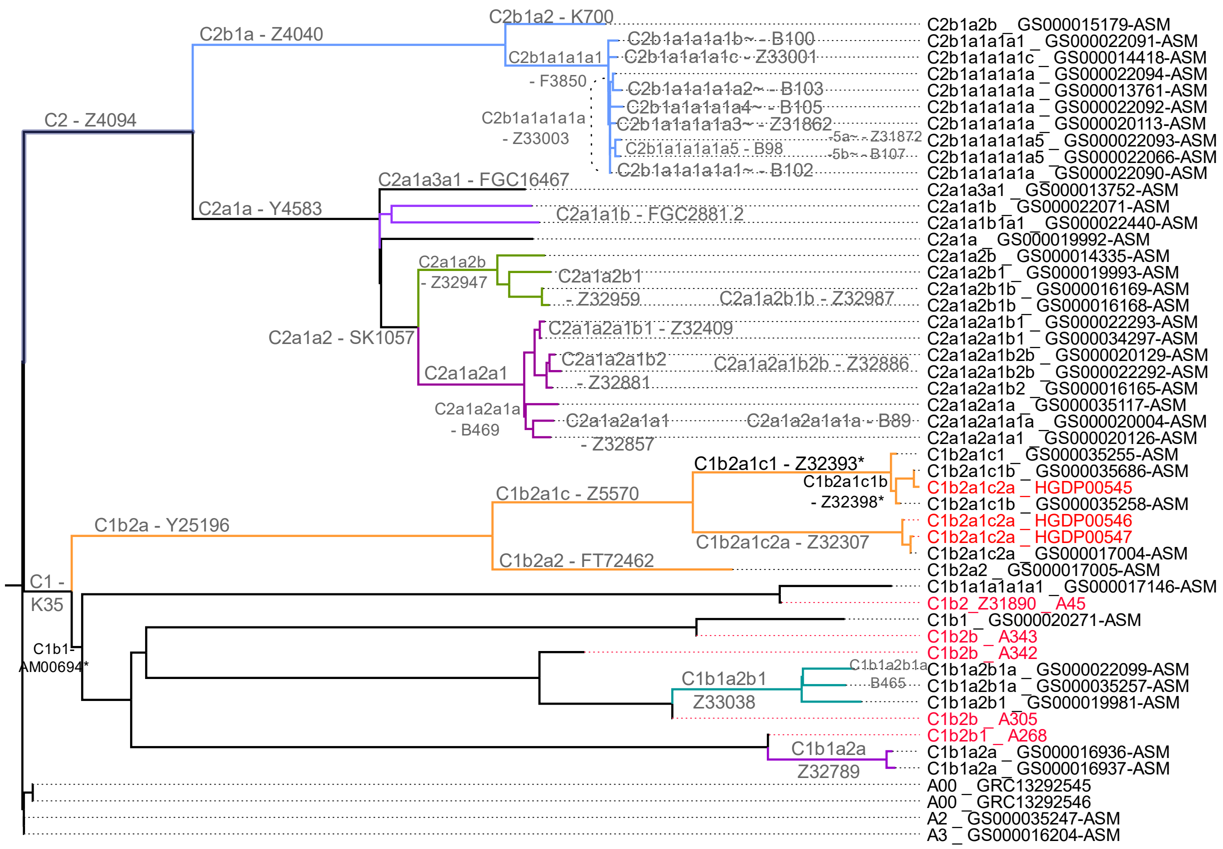

Supplementary Figure 3: Maximum likelihood phylogeny of the Y-chromosomal clade C from testdata 2. The tip labels present one individual each and are composed of the highest resolution (or most downstream) clade and the sequence name. Branches that are highlighted in colors present clearly separated sub-lineages. One SNP shared only by sequences of certain sub-lineages is labeled at the respective clade branches or nodes. A total of eight sequences with a missing data fraction of 76% (instead of the usual 20% per sequence) were marked in red. SNPs were exclusively reported in sequences of a clade, except for the red-marked sequences. In these cases, the SNP label is followed by an asterisk. The tree was manually rooted with four ancestral outgroup sequences.

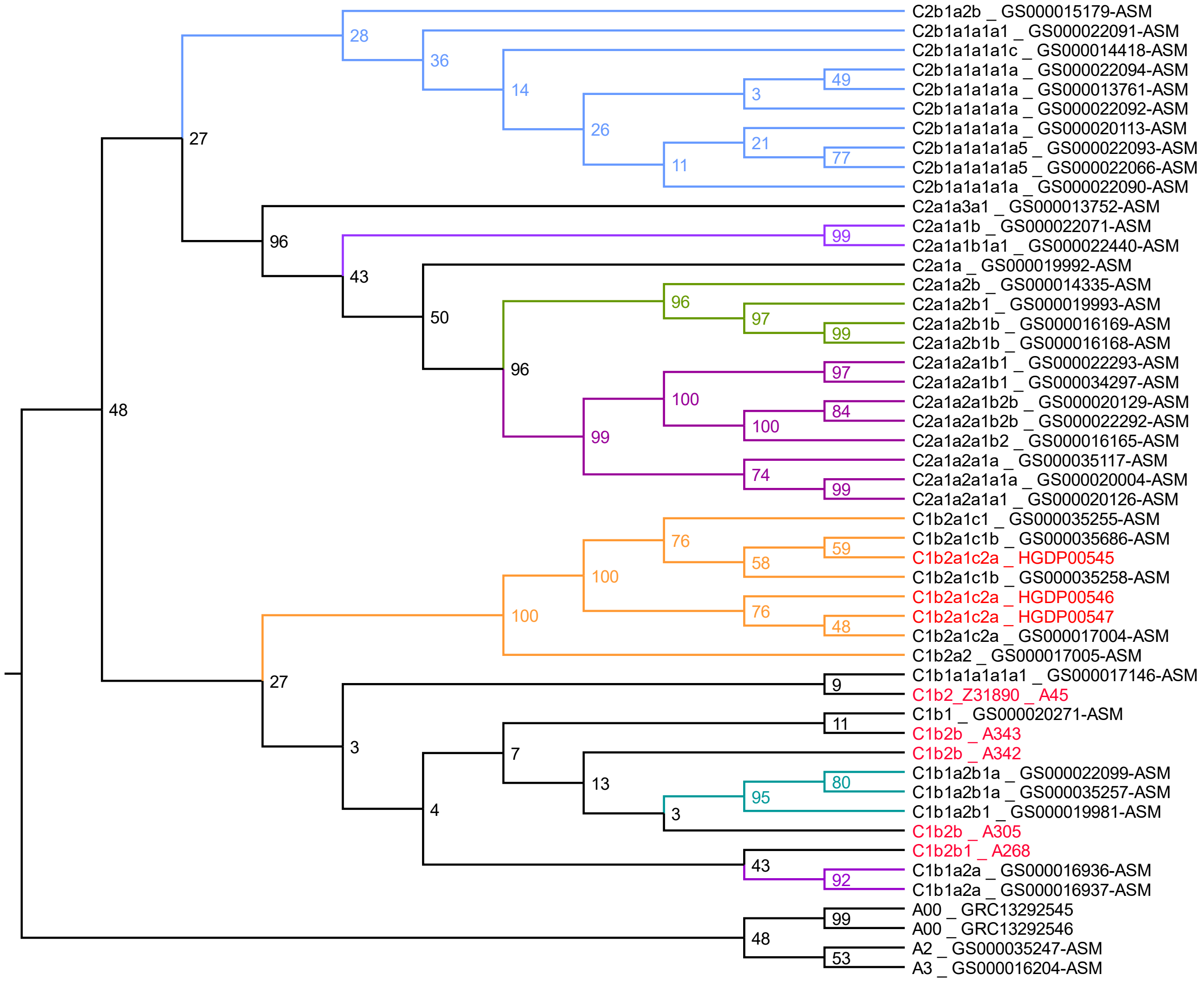

Supplementary Figure 4: Maximum likelihood phylogeny of the Y-chromosomal clade C from testdata 2 shown as a cladogram to present the bootstrap values.

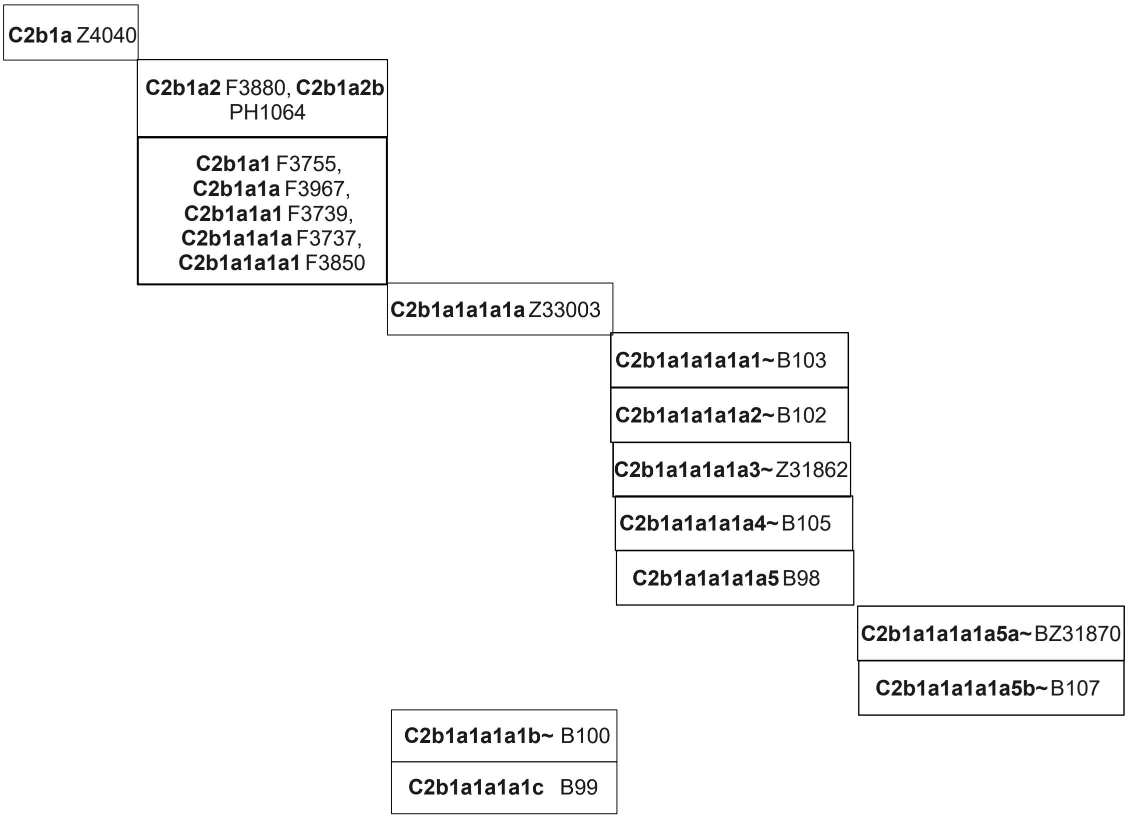

Supplementary Figure 5: Phylogeny of clade C2b1a from dataset 2 as an example of a clade with moderate bootstrapping values in a ML-based phylogenetic tree. The same phylogeny was obtained using softwares SNPtotree v1.0 and RAxML v8.2.12.

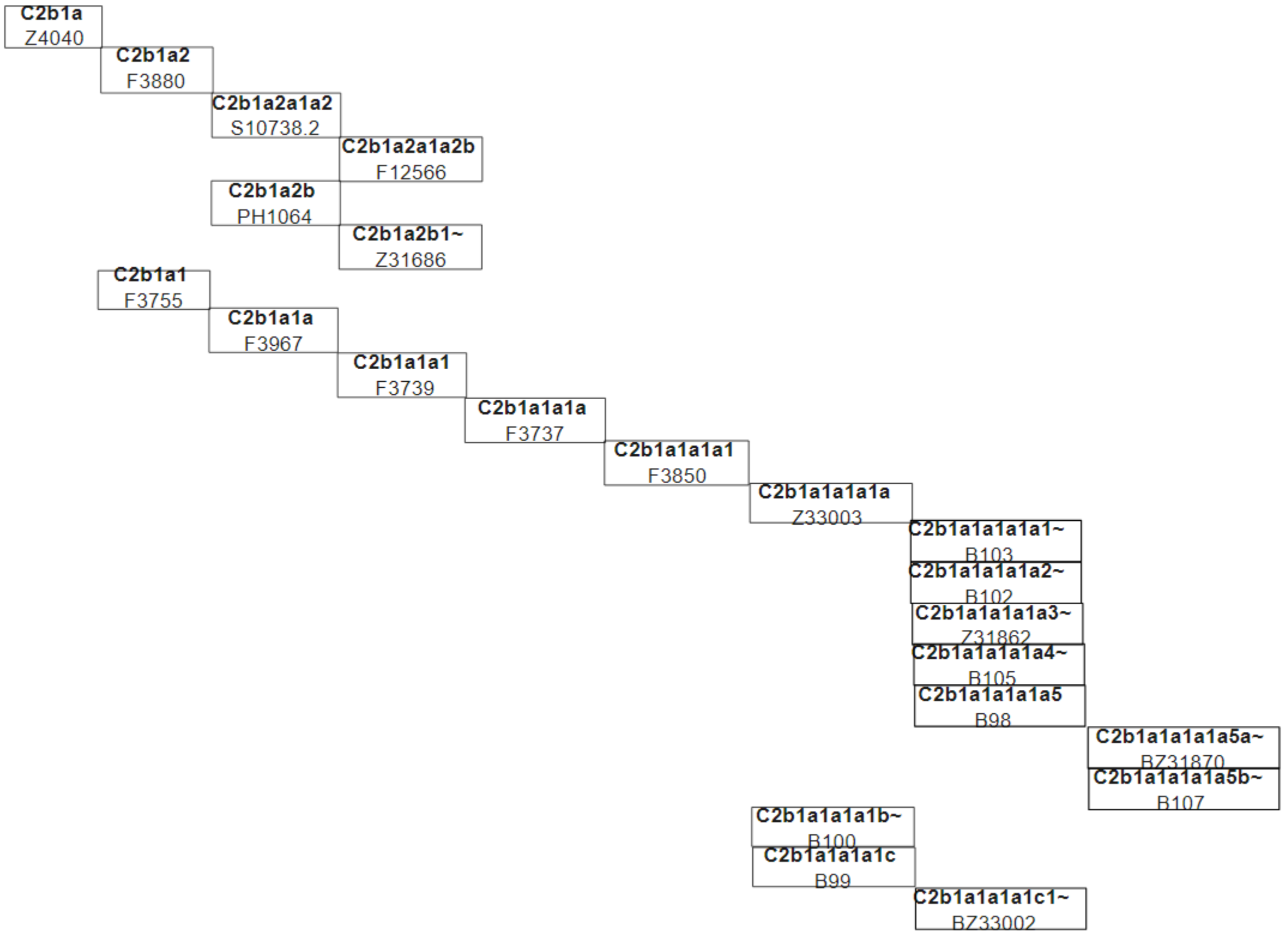

Supplementary Figure 6: True phylogeny of the already reported SNPs from the nested clade C2b1a from dataset 2 (taken from ISOGG Y-DNA Haplogroup Tree 2019-2020). For each clade, only one SNP is reported for simplification of the tree.
